# Smart Industrial IoT Monitoring and Control System Based on UAV and Cloud Computing Applied to a Concrete Plant

**DOI:** 10.3390/s19153316

**Published:** 2019-07-28

**Authors:** Marouane Salhaoui, Antonio Guerrero-González, Mounir Arioua, Francisco J. Ortiz, Ahmed El Oualkadi, Carlos Luis Torregrosa

**Affiliations:** 1 Department of Automation, Electrical Engineering and Electronic Technology, Universidad Politécnica de Cartagena, Plaza del Hospital 1, 30202 Cartagena, Spain; 2 Laboratory of Information and Communication Technologies (LabTIC), National school of applied sciences of Tangier (ENSATg), Abdelmalek Essaadi University, ENSA Tanger, Route Ziaten, BP 1818, Tanger, Morocco; 3 DSIE Research Group, Universidad Politécnica de Cartagena, Plaza del Hospital 1, 30202 Cartagena, Spain; 4 FRUMECAR S.L., C/Venezuela P.17/10 Polígono Industrial Oeste, 30169 Murcia, Spain

**Keywords:** UAVs, drones, industry 4.0, concrete plant, IoT protocols, IoT gateway, image recognition, cloud computing, network latency, end-to-end delay

## Abstract

Unmanned aerial vehicles (UAVs) are now considered one of the best remote sensing techniques for gathering data over large areas. They are now being used in the industry sector as sensing tools for proactively solving or preventing many issues, besides quantifying production and helping to make decisions. UAVs are a highly consistent technological platform for efficient and cost-effective data collection and event monitoring. The industrial Internet of things (IIoT) sends data from systems that monitor and control the physical world to data processing systems that cloud computing has shown to be important tools for meeting processing requirements. In fog computing, the IoT gateway links different objects to the internet. It can operate as a joint interface for different networks and support different communication protocols. A great deal of effort has been put into developing UAVs and multi-UAV systems. This paper introduces a smart IIoT monitoring and control system based on an unmanned aerial vehicle that uses cloud computing services and exploits fog computing as the bridge between IIoT layers. Its novelty lies in the fact that the UAV is automatically integrated into an industrial control system through an IoT gateway platform, while UAV photos are systematically and instantly computed and analyzed in the cloud. Visual supervision of the plant by drones and cloud services is integrated in real-time into the control loop of the industrial control system. As a proof of concept, the platform was used in a case study in an industrial concrete plant. The results obtained clearly illustrate the feasibility of the proposed platform in providing a reliable and efficient system for UAV remote control to improve product quality and reduce waste. For this, we studied the communication latency between the different IIoT layers in different IoT gateways.

## 1. Introduction

The emerging “Industry 4.0” concept is an umbrella term for a new industrial paradigm which embraces a set of future industrial developments including cyber-physical systems (CPS), the Internet of things (IoT), the Internet of services (IoS), robotics, big data, cloud manufacturing and augmented reality [1]. Industrial processes need most tasks to be conducted locally due to time delays and security constraints, and structured data needs to be communicated over the internet. Fog computing is a potential intermediate software that can be very useful for various industrial scenarios. It can reduce and refine high volume industrial data locally, before being sent to the cloud. It can also provide local processing support with acceptable latency for actuators and robots in a manufacturing industry [2]. The lack of interoperability between devices in the Industrial Internet of things (IIoT) considerably increases the complexity and cost of IIoT implementation and integration. The search for seamless interoperability is further complicated by the long lifetime of typical industrial equipment, which require costly upgrades or replacements to work with the newest technologies [3].

One of the novelties of autonomous robots applied to industry 4.0 is the generalization of the use of drones (unmanned aerial vehicles—UAVs) to carry out a multitude of inspection and data collection tasks. In this paper, the focus is on the construction industry since it is one of the sectors where traditionally less advanced technology has been applied and is therefore suitable for the use of the new Technology of Industry 4.0 [4]. Construction companies have mostly been using UAVs for real-time jobsite monitoring and to provide high-definition (HD) videos and images for identifying changes and solving or preventing many issues [5]. They are also used for inspection and maintenance tasks that are either inaccessible, dangerous, or costly from the ground [6].

Integrating UAVs into the IoT represents an interoperability challenge, as every IoT system has its own communications protocol. Moreover, a small error or delay beyond the tolerated limit could result in a disaster for various applications, such as UAV and aircraft manufacture and monitoring. While the (IoT) provides Internet access to any ‘thing’, UAVs can also be part of these connected things and send their on-board data to the cloud [7]. The off-board base station gives them higher computational capacity and the ability to carry out more complex actions using high-level programming languages, or leveraging services from computer vision tools by acquiring, processing, analyzing and understanding digital images in real-time. Computing capabilities can be extended to the cloud, taking advantage of the services offered, and saving the cost and energy consumption of an embedded UAV system. There is a growing trend towards the three-layer IIot architecture with fog computing, with a convergence network of interconnected and distributed intelligent gateways.

Fog computing is a distributed computing paradigm that empowers network devices at different hierarchical levels with various degrees of computational and storage capacity [8]. In this context, fog computing is not only considered for computation and storage but also as a way of integrating the different new systems capable of interconnecting urgent and complex processing tasks. The fog can be responsible for technical assistance between humans and machines, information transparency, interoperability, decentralized decision-making, information security, and data analysis. Its notable benefits minimize human error, reduce human health risks, improve operational efficiency, reduce costs, improve productivity, and maintain quality and customer satisfaction [2].

Here, we propose a UAV-based IIoT monitoring and control system integrated into a traditional industrial control architecture by harnessing the power of fog middleware and cloud computing. The main aim of the work was to present an innovative concept and an open three-layer architecture, including a UAV, to enhance quality and reduce waste by introducing visual supervision through cloud services as part of the three-layer IIoT architecture with fog computing and a control system. We also analyzed the fog computing layer and the IoT gateways to comply with the requirements of interoperability and time latency. We developed a theoretical model to mathematically represent the end-to-end latency in UAV-based Industry 4.0 architecture. We provide a comparative study of a fog computing system through different platforms and analyze the impact of these platforms on the network performance. We also describe a case study in a bulk concrete production plant using a drone-borne camera and IBM Watson’s service image recognition in the cloud. The study involved monitoring the materials carried on conveyor belts and controlling the production process. This operation was considered as cost-effective and time effective and reduced the concrete batch production time.

The paper’s main contributions are as follows:

- A proposal for an IIoT-based UAV architecture for monitoring and improving a production process using cloud computing services for visual recognition.
- Assessment of the three-layer architecture latency.
- Practical implementation and validation of the proposed architecture.

## 2. Related Works

The Industry 4.0 concept was born to apply the ideas of cyber-physical systems (CPSs) and IoT to industrial automation and to create smart products, smart production, and smart services [9]. It involves cyber-physical systems, the Internet of things, cognitive computing and cloud computing and supports what has been termed a “Smart factory”. In 2011, Germany adopted the idea to develop its economy in the context of an industrial revolution with new technologies compatible with old systems [10]. Industry now faces the challenge of making the IT network compatible with its machines, including interoperability, fog/cloud computing, security, latency, and quality of service. One of the proposed solutions is smarter IoT gateways [11], which are the bridges between the traditional network and sensor networks [12]. An IoT gateway is a physical device with software programs and protocols that act as intermediaries between sensors, controllers, intelligent devices, and the cloud. The IoT gateway provides the necessary connectivity, security, and manageability, while some of the existing devices cannot share data with the cloud [13].

EtherCat, CANOpen, Modbus/Modbus TCP, EtherNet/IP, PROFIBUS, PROFINET, DeviceNet, IEEE802.11, ISA100.11a, and Wireless HART are the most frequently used industrial protocols [14]. Due to the incompatible information models for the data and services of the different protocols, interoperability between the different systems with different protocols is always difficult. Up to only a few years ago the communication systems for industrial automation aimed only at real-time performance suitable for industry and maintainability based on international standards [15]. The Industry 4.0 concept has the flexibility to achieve interoperability between the different industrial engineering systems. To connect the different industrial equipment and systems, the same standards and safety levels are required. Open Platform Communications Unified Architecture (OPC UA) is a machine-to-machine (M2M) communications protocol developed to create inter-operable and reliable communications and is now generally accepted as standard in industrial plant communications [16]. OPC UA is an independent service-oriented architecture that integrates all the functionality of the individual OPC Classic specifications into one extensible framework [17]. Girbea, et al. [18] designed a service-oriented architecture for the optimization of industrial applications, using OPC UA to connect sub-manufacturing systems and ensure real-time communication between devices.

OPC UA can allocate all manufacturing resources, including embedded systems, to specific areas and extensible computing nodes through the address space and a pre-defined model. It solves the problem of unified access to the information of different systems [19]. Infrastructure protocols have been proposed in many studies; for example, the authors of [19,20] developed an edge IoT gateway to extend the connectivity of MODBUS devices to IoT by storing the scanned data from MODBUS devices locally and then transferring the changes via an MQTT publisher to MQTT clients via a broker. In [21], MQTT was adopted for machine-to-machine (M2M) communications to complement the MODBUS TCP operations in an IIoT environment. This environment integrates the MQTT event-based message-oriented protocol with the MODBUS TCP polling-based request–response protocol for industrial applications. The authors of [22] designed and implemented a web-based real-time data monitoring system that uses MODBUS TCP communications in which all the data are displayed in a real-time chart in an Internet browser, which is refreshed at regular intervals using HTTP polling communications. The success of the IIoT initiative depends on communication protocols able to ensure effective, timely and ubiquitous aggregation [23].

Implementing an Industry 4.0 architecture requires integration of the latest technologies, for example, IIoT, cyber-physical systems, additive manufacturing, big data and data analytics, cyber-security, cloud and edge computing, augmented and virtual reality, as well as autonomous robots and vehicles [24]. The cloud robotics architecture is based on two elements: the cloud platform and its associated equipment and the bottom facility. Bottom facilities usually encompass all kinds of mobile robots, unmanned aerial vehicles, machines, and other equipment [25]. The next generation of robots will include interconnected industrial robots [26], cobots [27] and autonomous land vehicles (AGVs) [28]. Cobots support human workers in various tasks, while robots can carry out specific tasks, such as looking for objects or transporting tools. UAVs and drones are among the emerging robot technologies that leverage the power of perception science and are now the preferred remote sensing system for gathering data over long distances in difficult-to-access environments [29]. Drone cameras can collect remotely sensed images from different areas safely and efficiently.

UAVs can save time and money in different sectors, such as agriculture, public safety, inspection and maintenance, transportation and autonomous delivery systems. This technological revolution was conceived to make people’s lives easier and to provide machine-to-machine communications without human intervention [30]. Many industries use drones or unmanned aerial vehicles to increase sensing and manipulation capabilities, autonomy, efficiency, and reduce production costs. In the construction sector, drones play a significant role in industrial sites; they can fly over and monitor an area by acquiring photos and videos. They can be used to check a given installation or production areas, to transmit data, monitor construction processes, and detect anomalies.

As mentioned in [4] many applications have already been implemented in the construction and the infrastructure fields. The net market value of deploying UAVs in support of construction and infrastructure inspection applications accounts for about 45% of the overall UAV market. UAVs are also used for the real-time inspection of power lines. In [31], the authors implemented drones to detect trees and buildings close to power lines. They can also be deployed to monitor oil, gas and water pipelines. Industrial SkyWorks [32] employs drones for building inspections and oil and gas inspections in North America using the powerful machine learning BlueVu algorithm to process the data collected. They provide asset inspection and data acquisition, advanced data processing with 2D and 3D images and detailed reports on the property inspected.

Crack assessment systems for concrete structures are constantly improving thanks to computer vision technologies and UAVs. UAVs combined with digital image processing have been applied to crack assessment as a cost-effective and time-effective solution, instead of visual observation [33]. Image processing has become a significant asset for UAVs systems and not only in industry. Capturing footage and videos generates a huge amount of data, for which cloud computing is vital. Image recognition technology has a great potential in various industries and has been improved by deep learning and machine learning image recognition systems (TensorFlow, and MATLAB) or image processing techniques such as computer algorithms for digital image processing. In [34], Machine Learning Techniques were used to estimate Nitrogen nutrition levels in corn crops (Zea mays). The work described in [35] introduced a real-time drone surveillance system to identify violent individuals in public areas by a ScatterNet hybrid deep learning (SHDL) network. In [36], the images from a drone camera were processed by the bag-of-words algorithm to detect crops, soils and flooded areas, with MATLAB to program the feature extraction algorithm. In [37], a solution was proposed to detect a final target using the drone’s camera. The system implemented image processing algorithms using the open source computer vision library OpenCV. The main goal was to resolve the energy constraint without any wire connections or human intervention. Cloud solutions like Google AI, Amazon Web Services, and IBM Watson offer on-demand access to their image recognition services to connect with other systems in the internet. The authors in [38] propose to move computationally-demanding object recognition to a remote computing cloud, instead of implementing it on the drone itself, by means of a cloud-based approach that allows real-time performance with hundreds of object categories. Other cloud-based platforms, e.g., SenseFly [39], Skycatch [40], and DroneDeploy [41], offer their own end-to-end solution that incorporates mission control, flight planning, and post-processing. These solutions provide image analysis through a real connection with the main application.

**Figure 1:**
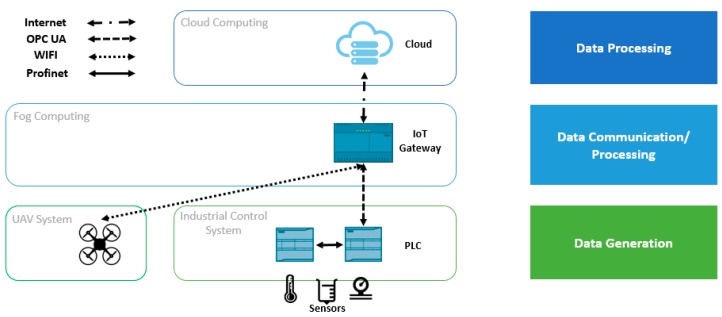
Proposed UAV-IIoT Platform.

The aforementioned studies show the significant advantages in different sectors of cost-effective and time-effective UAVs integrated with big data technology and machine learning. However, as far as we know, no studies have so far been published on the integration of UAVs into a complete industrial production system. Thus, here we propose an industrial real-time monitoring system with UAVs, fog computing and deep learning in the cloud (Figure 1). The proposed IIoT-based UAVs collect photos from an industrial plant, while the cloud processing platform analyzes them and sends the results to a control system.

## 3. Industrial IoT Monitoring and Control Platform

Industry is taking advantage of ever more complex and sophisticated systems. Systems not designed to communicate across production lines often require integration with pre-existing devices. The challenge of interoperability is thus one of the main concerns in designing intelligent human-to-machine and machine-to-machine cooperation. Ensuring systems-of-systems communications involves blending robotics, interconnected devices/sensors, actors, heterogeneous systems, and convergent hybrid infrastructure with IIoT and CPS systems, including fog/edge computing and cloud services. Our aim was to design a drone-based monitoring system able to interact in real-time with industrial sensors, PLCs, and the cloud automatically via an IoT gateway as middleware, and to transmit data between the different systems securely. We validated our proposed architecture in an industrial concrete production plant in a case study to improve production and reduce costs.

### 3.1. Proposed Platform/Architecture

A UAV monitoring system was elaborated as an industrial control system to reduce inspection time and costs. An overview of the approach can be seen in Figure 1. The proposed IIoT architecture is divided into three layers, with the UAVs in the data generation layer. The first layer consists of an industrial control system connected to a central collection point, which is the IoT gateway. The second layer is the fog computing layer for computation, storage, and communications. The last layer is a cloud back-end with image processing techniques. The fog layer connects the industrial control layer to the UAV system, the UAV system to the cloud, and finally the cloud to the industrial control system.

The control system receives data from remote or connected sensors that measure the process variables’ (PVs) setpoints (SP). When the system detects a trend change between PVs and SP, the change is routed to the programmable logic controllers (PLCs) and the central point (IoT gateway) to trigger the UAV system’s reaction. In this case, the human operator is replaced by a remote cloud calculation algorithm and a UAV system, in the sense that the UAV’s front camera serves as an additional surveillance sensor that is processed in the cloud to imitate an operator’s visual inspection. The drone goes to a specific point to supervise the process using the front camera. The UAV system is triggered automatically by responding to the sensor data from the industrial control system and data analyzed in the IoT gateway. The IoT gateway receives the captured photos and sends them to the cloud, which adopts deep learning techniques to analyze and send the results to the IoT gateway and the control system to confirm the anomaly.

### 3.2. IoT Gateway Capabilities

The IoT gateway is able to connect the sensor network to the cloud computing infrastructure and perform edge and fog computing and serves as a bridge between sensor networks and cloud services. Experiments were carried out using Node-RED and Ar.Drone library [42] to connect to the industrial control system, the cloud, and the UAV. Node-RED is a programming tool for wiring together hardware devices, APIs, and online services using JavaScript runtime Node.js and a browser-based editor. It controls the flows to be designed and managed graphically. Node-RED has a sample set of nodes for communications between different protocols and platforms. Node.js is considered one of the best platforms to build real-time, asynchronous and event-driven applications [15,43,44]. The Ar.Drone library [42] is an application also developed in Node.js and implements the networking protocols used by the Parrot AR Drone 2.0 [42]. This library provides a high-level client API that supports all drone features and enables developers to write autonomous programs. Using this library, the drone can be controlled via Wi-Fi, and automatically moves to a given target. It is also possible to describe the path, height, and direction the drone must follow to take the required photos.

### 3.3. The UAV-IIoT Architecture Development

This section describes the development of the proposed IIoT-UAV control system and its network protocols. It contains three layers, namely the industrial control system and UAVs, the IoT gateway, and the cloud. In the first layer, the industrial sensors of the control system are connected to a PLC that acts as OPC UA server, which routes the sensor data to the IoT gateway, which incorporates an OPC UA client installed in Node-RED. With the OPC UA client-server, data communication is independent of any particular operating platform. The central layer of the architecture augments the processing and communication abilities in the IoT gateway by connecting to the control system and cloud services, this part is considered as fog computing and depends on the sensor data retrieved from the sensors and driven to the OPC UA client node.

**Figure 2:**
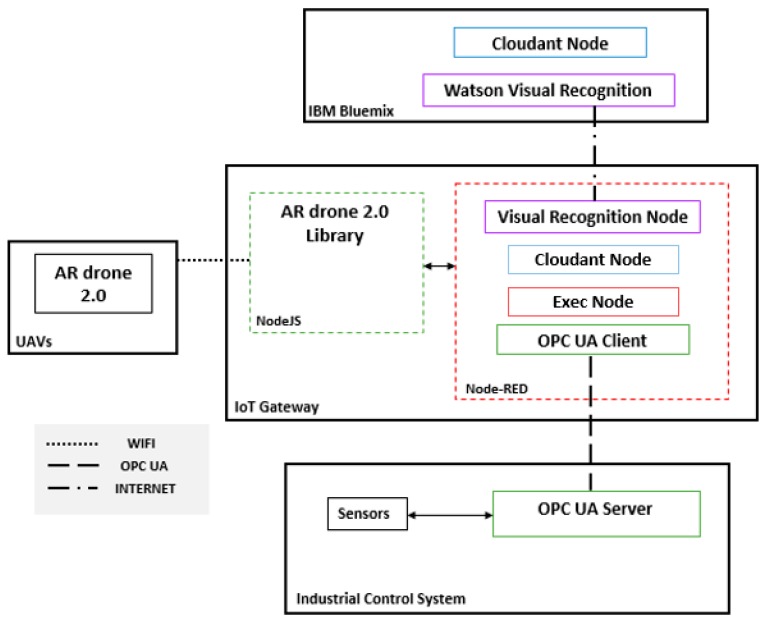
Development design of autonomous IIoT flight.

The fog layer is responsible for communications between all the other layers; it takes decision automatically based on the results and data received and conveys the output to the other layers or applications. The fog layer is presented in Figure 2 as an IoT gateway, which can support all the necessary tools and protocols to ensure communication storage and computing. Node-RED is considered the key programming tool for wiring together the industrial control system, UAV applications, and the cloud. Node-RED makes it easy to wire together flows using a wide range of nodes.

The main nodes in this case study are the visual recognition node, OPC UA client, Cloudant node and Exec node. In Figure 2, Node-RED is connected to the other systems and applications. Node-RED can connect to the Node.js Ar.Drone library in the IoT Gateway using the Exec node. While carrying out the mission triggered from Node-RED, the drone takes the necessary photos and sends them to the IoT gateway, in which Node-RED connects them to the Watson Visual recognition (WVR) node, which uses Watson visual recognition in the IBM cloud. The WVR node identifies the types of material transported on the conveyor belts and classifies the images according to the trained custom model. The photos are then sent to the IBM cloud by the Cloudant node, which is connected to the Cloudant database in the IBM. These photos can also be requested at any time by the Cloudant node in Node-RED (Figure 2).

By implementing an MQTT client library in Node.js, MQTT messages can be used to send commands to the drone through a MQTT broker installed in the cloud and also request Navigation Data (NavData) from the drone, such as battery life, wind-speed, and velocity. MQTT can also be used as an alternative or supplement to the OPC UA protocol in the industrial control system. The focus of the present paper is to evaluate the proposed approach using only the OPC UA protocol.

**Figure 3:**
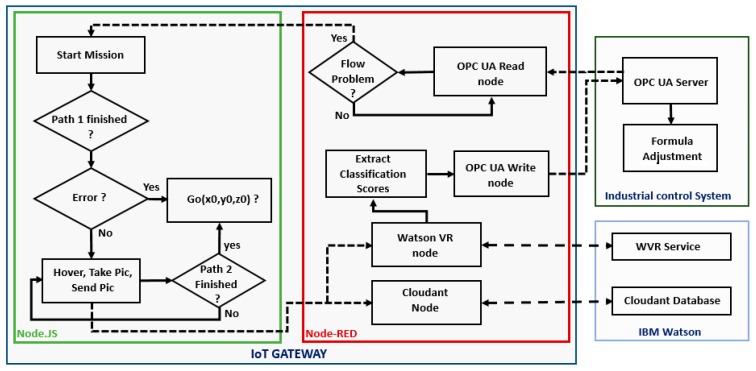
Communication process in the fog layer.

**Figure 4:**
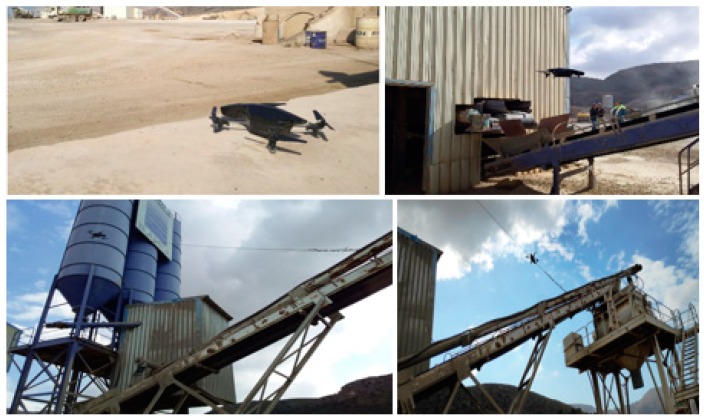
An AR.Drone 2.0 mission in the concrete plant.

Figure 3 details the communication process between the different parts of the proposed approach, including data flows between the different nodes, the industrial control system, UAVs and the cloud. Two main applications are installed in the IoT gateway: the Node-RED application and the Node.js application. The former facilitates communications, while the latter controls the drone. Node-RED checks the flow by reading the data from the OPC UA node, which is connected to the automation control system. If a problem is confirmed from the PLC, Node-RED triggers the drone mission executed by Node.js. The drone mission (Figure 4) is split into three paths: planning the mission, taking photos, and returning to the starting point. The Watson visual recognition node and Cloudant node receive the images and send them to the IBM cloud for processing and storage. The visual recognition node then forwards the results to the plant control system.

**Figure 5:**
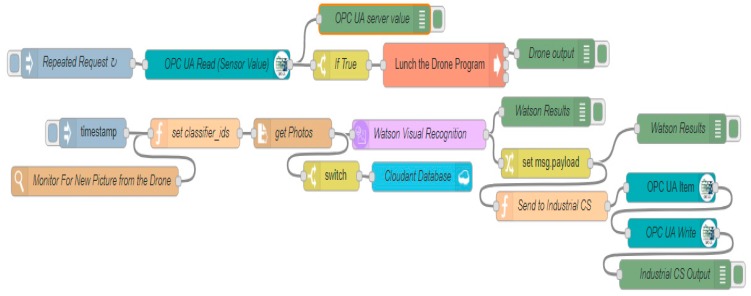
Node-RED flow of the IoT gateway with the path from PLCs to drone, drone to Watson, and Watson to the plant control.

Figure 5 shows the flows used in Node-RED in the IoT gateway. The OPC UA node is responsible for reading the updated data from the PLC and sending the results to the Exec node to launch the UAV mission. After the mission, the drone photos are saved in a folder on the IoT gateway by the watch node that monitors all new photos and sent to Watson’s visual recognition node for processing. The cloud visual recognition service analyzes the photos and classifies them into two classes. Each WVR result is provided as a score between 0.0 and 1.0 for each image for each trained class. The IoT gateway then receives the classification scores via the Watson VR node, the images’ scores are compared by the function node and the results are forwarded to the industrial control system and the PLC via the OPC UA write node for decision making.

### 3.4. UAV Mission Planning

The drone takes off at position (x,y), climbs to a certain altitude, hovers, returns to the start, and lands. The autonomous flight library was based on the AR.drone library [42], which is an implementation of networking protocols for the Parrot AR Drone 2.0. This library has four features: an extended Kalman filter, camera projection, and back-projection to estimate distance to an object, a PID Controller to control drone position, and a VSLAM to improve the drone position estimates [45,46].

The AR.Drone 2.0 is equipped with sensors with precise controls and automatic stabilization features, two cameras, a 60 fps vertical QVGA camera for measuring ground speed and a 1280 × 720 at 30 fps resolution front camera with a 92° (diagonal) field of view, Ultrasound sensors to measure height, three-axis accelerometer with +/−50 mg precision, three-axis gyroscope with 2000°/s precision, three-axis magnetometer with 6° precision, and a pressure sensor with +/−10 Pa precision. The drone can monitor its own position and mapping (SLAM), robustness and controls.

### 3.5. Case Study

**Figure 6:**
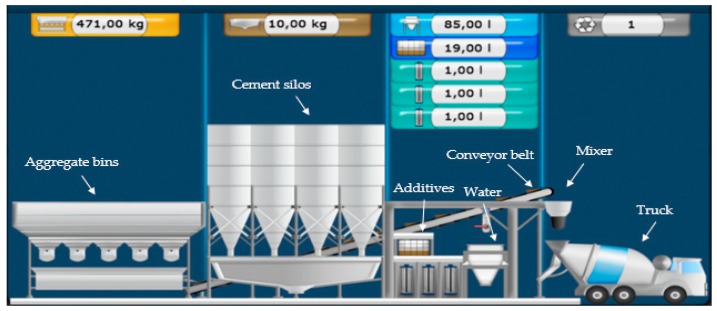
SCADA Industrial concrete plant with a typical concrete formula.

Concrete batching plants form part of the construction sector. Their many important components include cement and aggregate bins, aggregate batchers, mixers, heaters, conveyors, cement silos, control panels, and dust collectors. Concrete plants involve a human–machine interaction between the control system and the operator. The operator introduces the concrete formula by selecting the quantities of materials to be mixed and this data is processed by a control system so that the correct amount of material is conveyed to the mixer (Figure 6). The materials used in the concrete plant are aggregates, cement, admixtures, and water. The quality and uniformity of the concrete depend on the water-cement ratio, slump value, air content, and homogeneity.

Traditionally, to control concrete quality, microwave sensors are used in aggregate bins to measure the aggregate water content and then adjust the formula as required. Aggregates of different sizes are stored in bins for different formulas. Due to certain errors during the discharge and filtering process, these materials are sometimes mixed together incorrectly, affecting concrete quality and consistency.

The UAV camera and the service IBM WVR in the cloud can identify the state of the aggregate materials transported on the conveyor belts to make adjustments to the production process.

We use the cloud service to classify normal and mixed aggregates. The role of the drone in this case is to take pictures when the materials are being transported on the belts before they reach the mixer. The cloud classifies each image and returns the results to the IoT gateway as a score between 0.0 and 1.0 for each class. This result is sent to the PLC via the IoT gateway. Using these scores, any excess quantity of a material can be measured, and the required adjustments can be made to achieve the final formula. This operation eliminates wasted time and achieves the desired formula before the final mixing.

Drones are flexible, easy to deploy, can quickly change their position in a time-sensitive situation, and can be quickly configured. Incorporating them in a control system speeds up the production line by responding in real-time to the different requirements of the control system using the cloud services. The proposed approach is considered a cost-effective solution and replaces unnecessary and repeated operator controls, traditional monitoring, and control systems.

## 4. Delay Assessment in the Proposed Platform

One of the important challenges to overcome is the high-latency and unreliable link problems between the cloud and the IIoT terminals. Fog computing extends computing and storage to the network edge and is not only considered for computation and storage, but also as a way of integrating new systems capable of interconnecting urgent and complex processing systems. However, each fog and edge application may have different latency requirements and may generate different types of data and network traffic [47]. In this section, we focus on the latency between the data generation layer and the data communication layer (Figure 1). The data generation layer is composed of the UAV system and the industrial control system.

### 4.1. Industrial Control System Architecture

**Figure 7:**
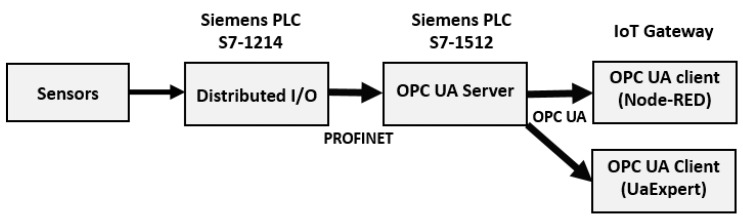
Functional description of the proposed architecture.

Figure 7 shows the proposed approach system for data collection and the first layer control between the sensors in the concrete plant. The sensors are connected to the PLC S7-1214 and all information for these sensors is sent from PLC S7-1214 to PLC S7-1512 using the industrial communication standard PROFINET over Ethernet. The PLC S7-1512 supports OPC-UA, which adopts client-server architecture. The OPC UA client is installed in the IoT gateway using the Node-RED OPC UA node. UaExpert is used in this case to check connectivity with the server. All the incoming information is controlled by Node-RED.

In this first part of the delay analysis, our focus will be only on the OPC UA communications between the IoT gateway and the PLC with the OPC UA server.

### 4.2. Latency between Two Terminals

**Figure 8:**
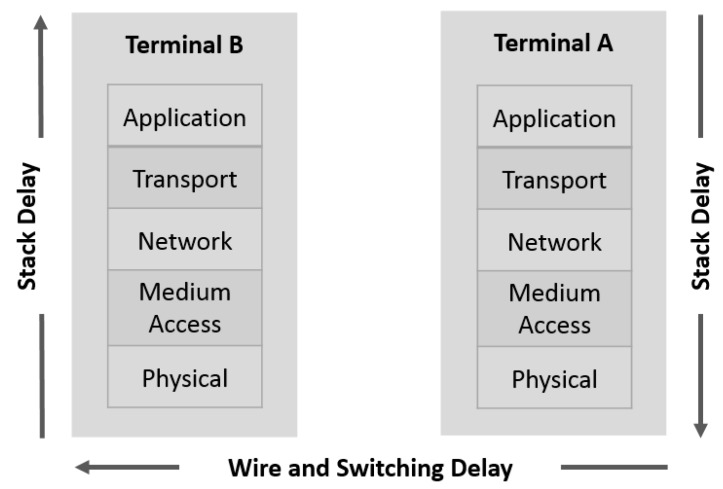
Latency between two terminals in a network.

Latency is the time network traffic delayed by the system processing, or the total time needed to send a network packet from the application on one server to the application on another server through the network interface controller (NIC), network (cable, Wi-Fi etc.), second NIC, and into an application on another server (or client). To assess the latency between two terminals, most approaches use the round-trip delay time (RTD) or the one-way delay (OWD). The latency in the context of networking is the time spent by propagation through the network support and hardware of the adapter, as well as the software execution times (application and OS) (Figure 8).

The hardware latency inside switches and on wires can be easily identified from the switch specifications, length of the wires, and the maximal transmission data rates, while the software latency imposed by processing a packet in the software stack is more arduous to evaluate. Several parameters like system workload, operating system and executed application influence software latency.

Equation (1) defines the RTD between two terminals in a network, where *t_A_* and *t_B_* are the software latency of the terminals *A* and *B* respectively, and *t_H_* marks the hardware latency of switches and wires connecting the terminals *A* and *B*.

(1) $$RTD=2.OWD=2.t_{A}+2.t_{H}+2.t_{B}$$

To accurately calculate OWD (by dividing the round-trip time by two), the configuration of the test systems must be perfectly symmetrical, meaning they must be running the same software, using the same settings, and have equal network and system performance.

### 4.3. Latency in OPC UA Network

**Figure 9:**
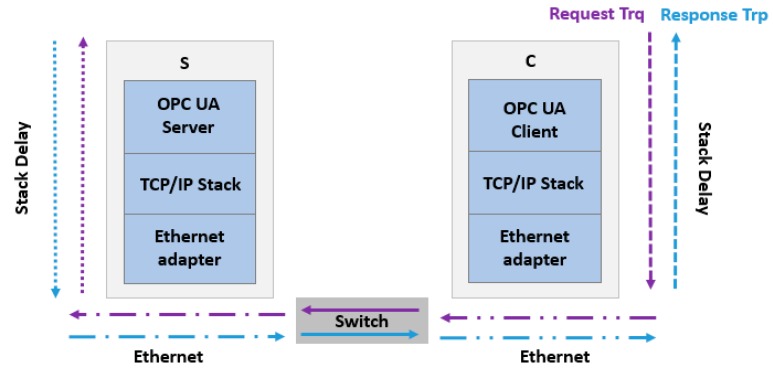
OPC UA delay in OPC UA client server in an Ethernet network.

In this section, we analyze the delays involved in client-server OPC UA communications in a switched Ethernet network. This model serves to define in detail the non-deterministic sources of end-to-end delay. The proposed model is based on time delays defined in [48,49] in an Ethernet-based network. Figure 9 shows the round-trip data path from an OPC UA server in PLC automate to an OPC UA client on the IoT gateway and the hardware OWD required.

We consider the end-to-end network delay in the switches and wires from the client request to the server, which can be divided into three categories, the frame transmission delay (*d_t_*), the time required to transmit all of the packet’s bits to the link, the propagation delay (*d_l_*), the time for one bit to propagate from source to destination at propagation speed of the link, and the switching delays (*d_s_*), which depend on the route through the network to the server.

The transmission delay depends on the length of packet *L* and capacity of link *C*. The propagation delay is related to the distance between two switches and the propagation speed of the link *S*.

(2) $$d_{l}=\frac{D}{S},d_{t}=\frac{L}{C}$$

The switch delay is defined as the time for one bit to traverse from switch input port to the switch output port. It is divided into four delays: the first is the switch input delay (*dS_in_*), the delay of the switch ingress port, including the reception of the PHY and MAC latency. The second is the switch output delay (*dS_out_*), the delay of the switch egress port, including the transmission PHY and MAC latency. The third delay is the switch queuing delay (*dS_q_*), the time a frame waits in the egress port of a switch to start the transmission onto the link. The last is the switch processing delay (*dS_p_*), the time required to examine the packet’s header and determine where to direct the packet is part of the processing delay.

(3) $$d_{S}\left(t\right)=d_{S_{in}}+d_{S_{p}}+d_{S_{out}}+d_{S_{q}}\left(t\right)$$

The hardware end-to-end delay *d_CS_* presented as a request from an endpoint server *S* to the destination endpoint in a client *C* can be expressed as the sum of the delays of all the switches and links in the path, *n* being the number of links and *n* − 1 the number of switches along the path.

(4) $$d_{CS}\left(t\right)=d_{t}+\overset{n}{\underset{i=1}{\sum }}\left(d_{l,i}\right)+\overset{n-1}{\underset{i=1}{\sum }}d_{s,i}\left(t\right)$$

**Figure 10:**
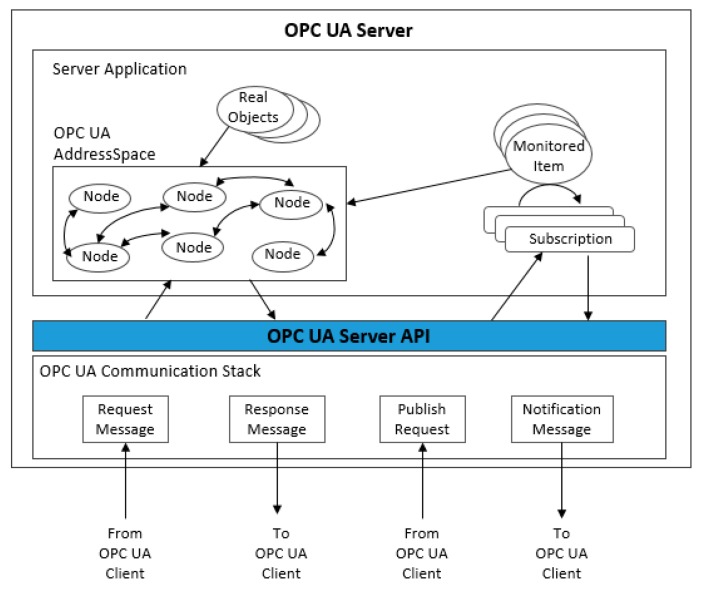
Architecture of the OPC UA Server.

Figure 10 reveals the architecture of the OPC UA server. The server application is the code that implements the server function. Real objects are physical or software objects that are accessible by the OPC UA server or internally maintained by it, such as physical devices and diagnostic counters. Particular objects, such as Nodes, are used by OPC UA servers to represent real objects, their definitions and references; all nodes are called AddressSpace. Nodes are accessible by clients using OPC UA services (interfaces and methods) [50].

In the case of *m* number of requests from clients to the nodes in the OPC UA server, the overall hardware end-to-end delay of the OPC UA client-server (*d_CS_*) communication over an Ethernet network, when there are *m* requests from the client to the server, is presented as:

(5) $$t_{H}=d_{CS}\left(t\right)=\overset{m}{\underset{j=1}{\sum }}\left(d_{t,j}\right)+\overset{n}{\underset{i=1}{\sum }}\left(d_{l,i}\right)+\overset{n-1}{\underset{i=1}{\sum }}\left(d_{s,i}\right)$$

By analyzing all the delays mentioned in the hardware, we admit that the end-to-end delay on Ethernet network is deterministic, except the delay in the switch queue, which depends on the link utilization. The packet queuing delay increases in a frequently used link.

By investigating the hardware delays for an OPC UA client/server communication in an Ethernet network, we conclude that it is hard to define exactly the hardware delay on the account of the queuing delay. In that case, when it comes to complex processes with real-time requirements, OPC UA reaches its limits. There are different ways of defining this delay, for example QoS techniques such as QFQ (weighted fair queuing) or strict priority [14]; however, there is always a certain delay and jitter that limits real-time performance. Time sensitive networking (TSN) provides mechanisms for the transmission of time-sensitive data over Ethernet networks. The adoption of OPC-UA over TSN will also drive this paradigm in the world of deterministic and real-time machine to machine communications. TSN provides mechanisms for the transmission of time-sensitive data over Ethernet networks. With Ethernet’s limitations in terms of traffic prioritization, the TSN working group has developed the time-aware scheduler (TAS), defined in 802.1Qbv [51]. TAS is based on TDMA, which solves the problem of synchronization and traffic priority in the Ethernet. By using this technique, queuing delay can be completely eliminated, hence the end-to-end latency becomes deterministic. Bruckner, et al. [52] adopted this method to evaluate OPC UA performance on TSN with the most commonly used communication technologies.

### 4.4. UAV System Delay

**Figure 11:**
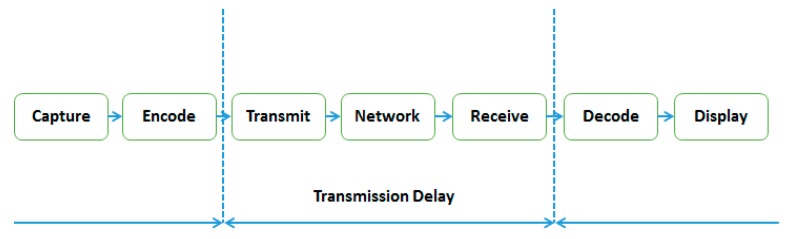
Video transmission system delay sources.

There are several ways to introduce latency in a drone’s video compression and transmission system. The end-to end delay in the system can be divided into seven categories (Figure 11): *T_cap_* is the capture time, *T_enc_* the time required to encode, the resulting transmission delay is *T_tx_*, *T_nw_* is the delay network when the drone is connected to the remote ground station via a network, *T_rx_* is due to the ground station also being wirelessly connected to a network, *T_dec_* is the decoding delay at the reception station, and *T_disp_* is the display latency.

(6) $$T=T_{cap}+T_{enc}+T_{tx}+T_{nw}+T_{rx}+T_{dec}+T_{disp}$$

Note that when the drone is communicating directly with the ground station, no network is involved and there is only a single transmission delay (*T_nw_* = 0 and *T_rx_* = 0).

(7) $$T=T_{cap}+T_{enc}+T_{tx}+T_{dec}+T_{disp}$$

In the H.264 system, each video frame is organized into slices which are in turn divided into non-overlapping blocks and macro-blocks (two-dimensional unit of a video frame). Every slice is independently encoded and can decode itself without reference to another slice. The main advantage of this system is that it is not required to wait for the entire frame to be captured before starting to encode. As soon as one slice is captured, the encoding process can start, and slice transmission can begin. This technique has a consistent effect on the overall latency as it influences all the system latencies from encoding to display.

Theoretically, we define the overall latency by the number of slices *N*, although in practice this may not be the case due to setting up and processing individual slices.

(8) $$T=T_{cap}+N.\left(T_{enc}+T_{tx}+T_{dec}+T_{disp}\right)$$

In order to efficiently transmit and minimize the bandwidth, it is important to use video compression techniques, although the slice technique also has an effect on the compression ratio. The higher the number of slices, the faster they can be encoded and transmitted, although as this number increases, the number of bits used for a slice and the effective slice transmission time also increase.

Other types of delay also affect the overall delay. Some factors can be adjusted when a UAV system is used. For example, *T_cap_* depends on the frame rate of the UAV camera; the higher the frame rate, the shorter the capture time. *T_x_* relies on the available data bandwidth of the transmission channel, while *T_disp_* (video capture) is based on the refresh rate of the display.

## 5. Drone Mission and IBM WVR Results

**Figure 12:**
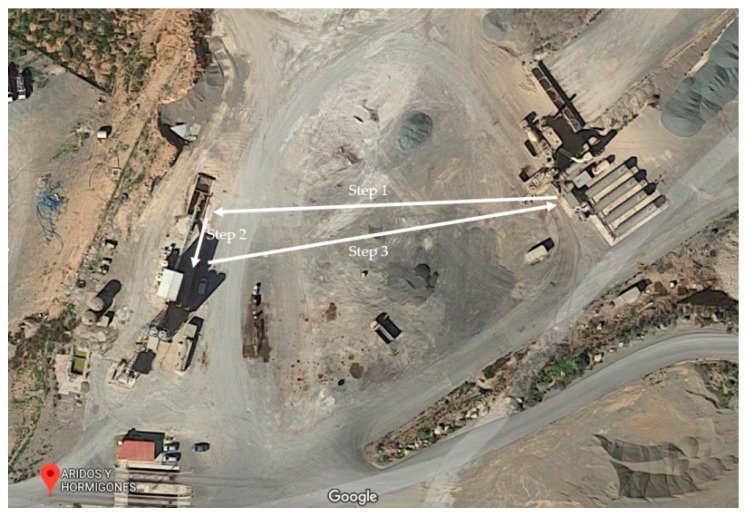
Path used by the drone to execute the mission in a concrete plant.

The drone in the worksite (concrete mixing plant) is located in the base station, which is at a distance from the conveyor belts and is always ready for new requests from the industrial control system. Using the library described in Section 3.4, the drone is able to automatically take-off and follow a predefined path around the conveyors belts to take the required photos (Figure 4). The drone’s mission is accomplished in three steps (Figure 12). The drone carried out 10 test missions in three days in a real concrete batching plant in Cartagena (Spain). The first step was to fly around 130 m to the beginning of the conveyor belts. It then hovered over the belts, took photos and sent them to the IoT gateway. In the last step the drone returned to the starting point (Figure 12).

### 5.1. IBM Watson Image Recognition Training

Off-board image processing techniques were selected due to the asset of the cloud services. MATLAB, OpenCV or TensorFlow could also have been used as the control system; however, the cloud completes the computing activities and provides an efficient time and cost optimization. IBM’s Watson visual recognition (WVR) service analyzes the content of images from the drone camera transmitted through the IoT gateway (see Figure 1). This service can classify and train visual content using machine learning techniques.

The WVR service enables us to create our own custom classifier model for visual recognition. Each sample file is trained against the other files, and positive examples are stored as classes. These classes are grouped to define a single model and return their own scores. There is also a default negative class to train the model with images that do not depict the visual subject of any of the other positive classes. Negatives example files are deployed to improve the results and are not stored as positives classes.

WVR is based in part on the technology developed for the IBM multimedia analysis and retrieval system (IMARS) [53], supplemented by “deep features” that are extracted on Caffe software [54]. The WVR service extracts feature vectors from a particular layer of a Caffe network for all the supplied examples and uses them to train a one-versus-all support vector machine (SVM) model for each class. The feature extraction process is therefore equivalent to “inferencing” with the neural network, but the SVM learning process is less CPU intensive than inferencing [55].

The Watson service generally accepts a maximum of 10,000 images or 100 MB per .zip file and a minimum of 10 images per .zip file, with different angles and scenarios to obtain the maximum precision. The service recommends that the images be at least 224 × 224 pixels and contain at least 30% of the subject matter.

**Figure 13:**
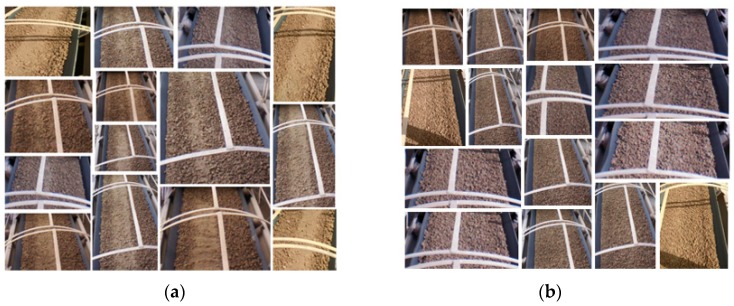
Dataset used to train the custom model in WVR service: (**a**) Shows images used to train the Mixed class; (**b**) Shows Images used to train the Normal class.

In order to train the custom model, we used a dataset of the images captured by the UAV camera from the field of practice in different positions. In addition, we roughly divided the use case into two parts: a mixed material set and a normal material set (Figure 13).

The classification is divided into two stages, the training stage and the testing and validation stage, and the images used in the second stage are not used in the first.

**Figure 14:**
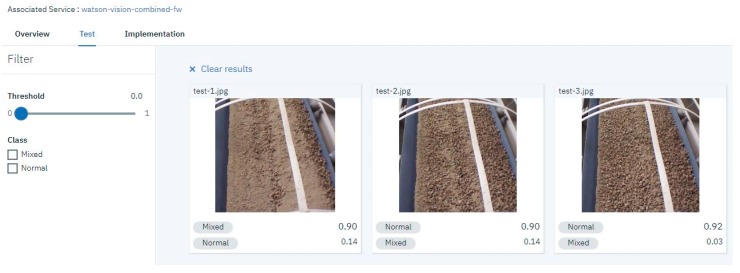
Watson visual recognition test of new images not used in the training phase.

In the training stage we used the dataset images to create two new classes, a Normal class, and a Mixed class. These classes were grouped to define a single custom model. In the testing stage, the results of the Watson tests are shown as a confidence score for the image in the range of 0 to 1. A higher score indicates that the class is more likely to be depicted in the image. The scores are considered as a threshold for action, and the confidence score are based on training images, evaluation images, and the types of criteria of the desired classification. Figure 14 shows the test of three different new images and the results of each class score. WVR recognized the difference between the images according to the density of the normal material on the conveyors. For instance, the confidence score for the test-3 .jpg image is 0.92 for the normal class, indicating the greater likelihood of this class being in the image.

### 5.2. WVR Performance Evaluation

To evaluate the performance of the WVR, we used a formula to calculate the accuracy as defined by Equation (9). To validate the WVR performance in this case, we tested a dataset of more than 100 photos and achieved a final detection accuracy of 87.28%. The misclassified cases are listed in Table 1, which represents the confusion matrix. Based on a large number of tests with new images not used in the training phase, we were able to define the threshold of each score class, make a decision, and then send the order to the industrial control system to adjust the material quantities on the conveyor belts.

(9) $$Accuracy=\frac{TP+TN}{TP+TN+FP+FN}$$

where *FP* represents the number of negatives samples that are judged to be positive, *TP* is the number of positive samples judged to be positive, *TN* the number of negative samples judged negative and *FN* is the number of positive samples judged to be negative.

### 5.3. IBM Watson Image Recognition Results

The novelty of the proposed IoT control system is that it provides real-time interaction between an industrial control system, UAVs and the cloud. Based on the input information from the concrete plant, the UAV can interact and execute the mission automatically and provide the necessary photos to the cloud to compute and analyze the data by deep learning methods and send the result back to the control system for decision-making.

**Figure 15:**
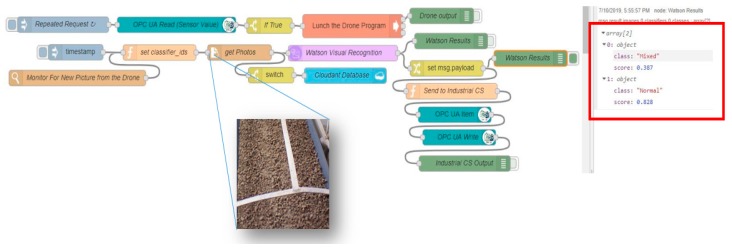
Node-RED flow and WVR results of a UAV photo.

After training the WVR model in the cloud, Node-RED can send new photos stored in the IoT gateway to the cloud service using the WVR recognition node and the get-file node (Figure 15). The cloud service classifies the new photos and sends back the results to the WVR node to be analyzed and sent to the PLC via the OPC UA protocol. Figure 15 shows the results obtained from the WVR node in Node-RED. The service classifies the image and produces two scores for each class.

## 6. System Performance

This section provides the RTD time measurements of the IoT gateway connections in its conditions of use and underlines the crucial role of the IoT gateway in terms of latency. In this application, the IoT gateway is connected to different systems with different transmitted data. Each IoT gateway has its own software and hardware components to process the data with different processing times. Below, we evaluate this difference by using three gateways with different performances. Table 2 shows the specification of each of the three selected platforms.

### 6.1. OPC Experimental Method and Results

**Figure 16:**
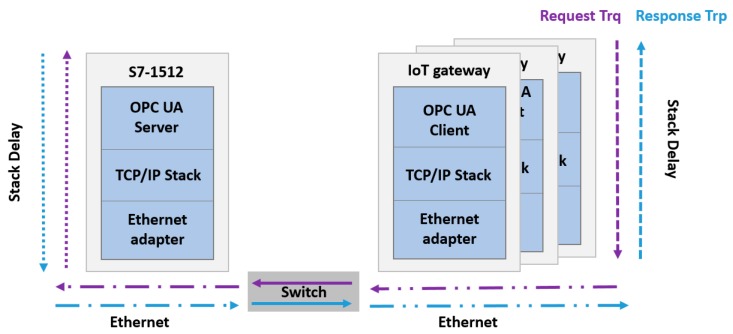
OPC UA delay in OPC UA client server in an Ethernet network.

A case study was used to define the latency of the OPC UA client-server architecture. The experimental set-up was based on an industrial plant and software in addition to three different IoT-based platforms. The industrial control system deployed as an OPC UA server uses a Siemens S7-1512 with embedded OPC UA communication stack. The OPC UA client is implemented using Node-RED OPC UA client node in the three different devices, the IoT gateway IOT2040 from Siemens (S-G), a Raspberry Pi 3 Model B (RPI-G) and a PC computer Toshiba SATELLITE (PC-G) (Figure 16). In the first step of the latency study we compared the *RTD* with the three different devices considered as OPC UA client attached to the same Siemens S7-1512 OPC UA server network.

The proposed application is deployed in a local network and is based on a simple use case that consists of reading one bit from the OPC UA server. All the RTD measurements were conducted on the same network. In these conditions we consider that RTD delay is derived mainly from the *Tx* software latency of the software stack of device *x* (Equation (1)), assuming insignificant hardware *T_H_* latency of the wires and switch.

A machine *M_X_* is defined as well as a pair of hardware setup *HW* and a software setup *SW*:

(10) $$M_{x}=\left(HW,SW\right).$$

The software setup *SW* is defined as the set of all software elements in this machine and the hardware setup *HW* is defined as the set of all hardware elements [56].

(11) HW={Memory,Processor,NIC…}

(12) SW={Application,OS,Drivers…}

**Figure 17:**
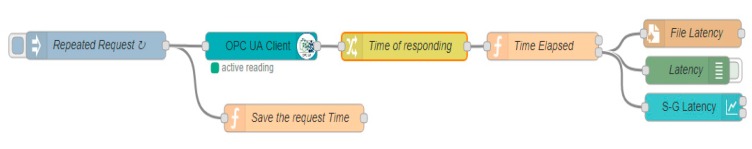
Node-RED flow used to calculate round trip latency (OPC UA Client to the OPC UA Server).

A timestamp contained in an inject node in Node-RED was used to measure latency (Figure 17). In every request, the timestamp request is saved by a function node. We define latency *L* as the difference between the timestamp of response from the server and the timestamp request of the client saved in the first function. Thus, latency *L* is measured as:

(13) $$L=T_{request}-T_{response}=RTD$$

**Figure 18:**
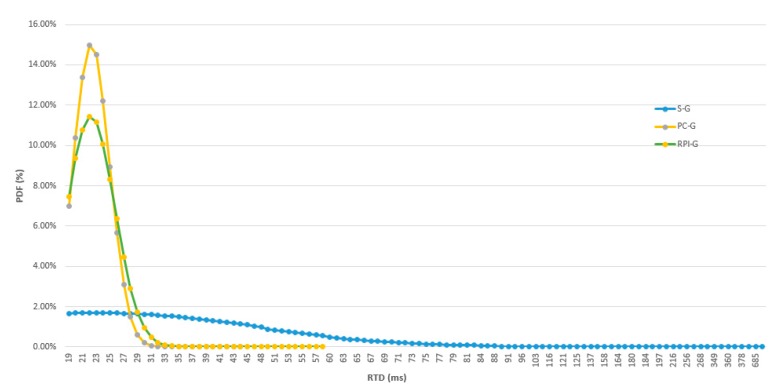
OPC UA client-server RTD to read one bit through different machines.

The latency results are summarized in Table 3, showing the *RTD* average, standard deviation, minimal and maximal values calculated for each fog computing machine. All the samples were thoroughly checked for the same architecture on different days in an experimental campaign with more than 5000 valid samples. The OPC UA requests were repeated each second to read the one bit value in the OPC UA server (Figure 17). S-G gateway latency is higher than in the RPI-G and PC-G gatways, approximately three times that of the RPI-G and seven times that of the S-G. This difference is evident in the probability density function as shown in Figure 18. The shapes of the RPI-G and the PC-G are almost the same with a single peak, while the S-G shape is narrower and scattered over a large time area.

**Figure 19:**
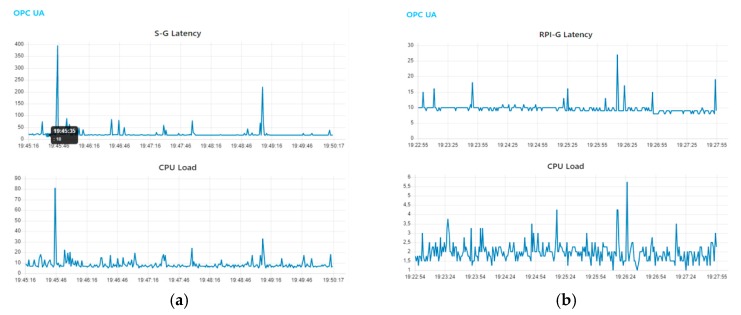
(**a**) Simulation results of CPU load (%) versus OPC UA RTD (ms) in the S-G; (**b**) Simulation results of CPU load (%) versus OPC UA RTD (ms) in the RPI-G.

In order to analyze this large difference in the recorded RTD between S-G and RPI-G, we continuously monitored the CPU load for 5 min during the OPC UA channel’s RTD. The S-G and RPI-G gateways were tested separately in the same network conditions and running only Node-RED, which runs the OPC UA client. The computed CPU usage was calculated as the average of all the cores in the S-G and RPI-G gateways (see Figure 19).

Given the analogy of a similar situation [57], which assumes that the larger the RTD pattern peaks the higher the probability they are due to the higher CPU load, although the recorded CPU load patterns are not only due to the OPC UA client implemented in Node-RED. Nonetheless, we compared the impact of CPU usage in the RTD as regards the same conditions in the two gateways. It should be noted that the impact of Node.js can be estimated to be around 10% of the processing power of the gateway used in the demonstration case, and the number of devices connected to the gateway linearly increases CPU and memory usage [15].

There is always intense use of CPU in the S-G RTD when high latency is detected. The S-G peaks sometimes exceed 400 ms (Figure 19 and Table 3) while in the RPI-G they do not exceed 80 ms. Furthermore, the average CPU load of the RPI-G is much lower than that of the S-G. The average value of the CPU load in the RPI-G is around 1.7%, while in the S-G it is around 8% and the number of devices connected to the gateway linearly increases the CPU load.

### 6.2. Watson Experimental Method

The IBM Watson visual recognition service is currently operated in a datacenter in Dallas (USA). Internally, the service uses deep neural networks to analyze images. More than one server is used to provide high throughput and reliability. Node-RED provides a node to connect to the WVR service that takes an image as input and produces a set of image labels as output.

The experiments carried out were based on Equation (13) and used the Node-RED flow. The latency results are summarized in Table 4, the RTD average, standard deviation, minimal and maximal values calculated for each fog computing machine. All the samples were carefully and thoroughly checked for the same architecture on the same day. The experiment was repeated for one sample field case image less than a data block size of 154,076 bytes. Each experimental campaign had around 100 valid samples for each machine. Between each 100 requests, the next request is triggered at the time of receiving the results of the previous request from the WVR.

The results reported in Table 4 display the differences between the different fog machines. The average S-G score is higher than RPI-G and PC-G. However, RPI-G is faster than S-G and had a larger standard deviation, while PC-G is faster than RSP-G with a low standard deviation.

**Figure 20:**
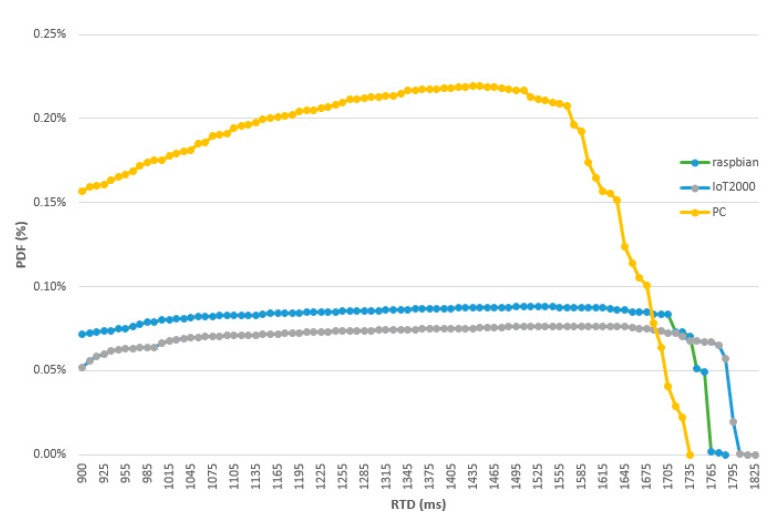
Probability density function estimates IBM WVR latency to classify an image located in the IoT gateway.

The probability density function estimates of the WVR delay for the three gateway machines are given in (Figure 20). In this case, the probability density of the RPI-G has almost the same curvature as that of S-G, while the probability density of PC-G is larger.

Since the WVR node in Node-RED relies on the HTTP protocol to send the images to the cloud, we performed another test using SpeedTest to measure HTTP throughput between the web server and client over the three gateways considered as clients (on the same day with the same network conditions). The results obtained in Table 5 present similar outcomes in the download, while the S-G upload is lower than the other gateways.

### 6.3. UAV Experimental Results

The streaming quality of the proposed Node.js application was measured under certain conditions of use to compare the response time on different IoT gateways in the same configurations and conditions. The transmission channel, frame rates and compression techniques were the same in all the tests on the recording of camera images and saving them to a folder in the IoT gateway. The image frames were captured and registered in a buffer before being sent to the gateway. Encoding was performed by FFMPEG codec, and the received frames were decoded in the gateway before being saved on the gateway disk.

#### 6.3.1. Codec Latency

The AR.Drone library [42] uses the basic H264 profile (MPEG4.10 AVC) for high quality streaming and video recording. The Baseline profile was targeted at applications in which a minimum of computational complexity and a maximum of error robustness are required. H.264/MPEG4-AVC baseline supports two slice-coding types. The simplest is the *I* slice, in which all macro-blocks are coded without referring to any other pictures in the video sequence. Previously coded images are used to form a prediction signal for macro-blocks of the predictive-coded *P* [58].

Theoretically, based on Equation (8), UAV delay can be estimated by:

(14) $$T=T_{cap}+2.\left(T_{enc}+T_{tx}+T_{dec}+T_{disp}\right)$$

#### 6.3.2. Experimental Results

The experiments focus on the mission delay generated by taking pictures in a concrete production plant. We measured the time needed for the drone to connect with the IoT gateway, take a picture and save it in a file in the IoT gateway (WriteFile function from Node.js). Table 6 shows the results of an AR.Drone 2.0 mission with around 200 images on the Node.js application, triggered from Node-RED. The latencies in both machines are expressed in milliseconds and calculated in the Node.js application.

Note that these tests were made with an image resolution of 640 × 360 px, frame rate of 5 fps and a codec with H264 baseline.

The results provided in Table 6 show the large difference in terms of latency between RPI-G and PC-G. The average RPI-G latency is almost three times that of PC-G, and RPI-G standard deviation is much higher than in PC-G.

On the other hand, the S-G results are consistently different from those of PC-G and RPI-G; the average S-G latency is very high, while the standard deviation is lower than RPI-G.

**Figure 21:**
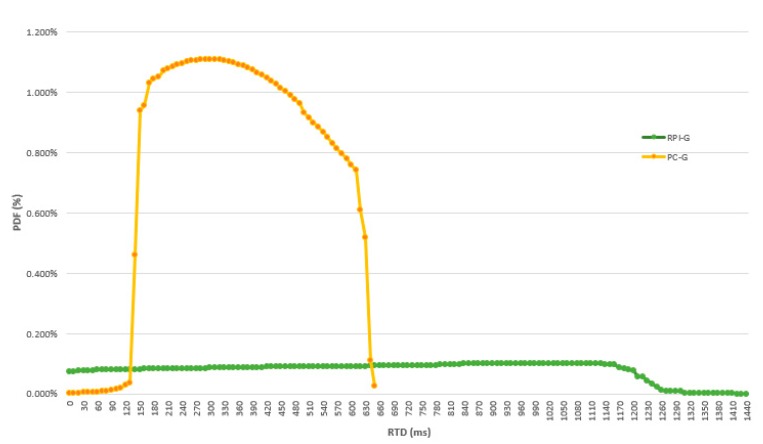
Probability density function of the delay of the drone connected to the gateway when successive pictures from PC-G and RPI-G are taken.

**Figure 22:**
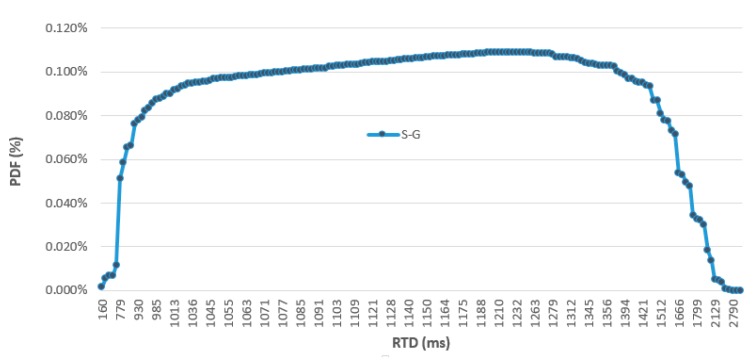
Probability density function of the delay of the drone connected to the gateway when successive pictures from S-G are taken.

The probability density function estimates of the WVR delay for the RPI-G and the PC-G are shown in Figure 21, while the probability density function estimates of the WVR delay for the S-G are shown in Figure 22. Here, the distributions are clearly different, the data spreading of the PC-G distribution covers a narrower range, with a larger spread in the RPI-G and S-G distributions.

**Figure 23:**
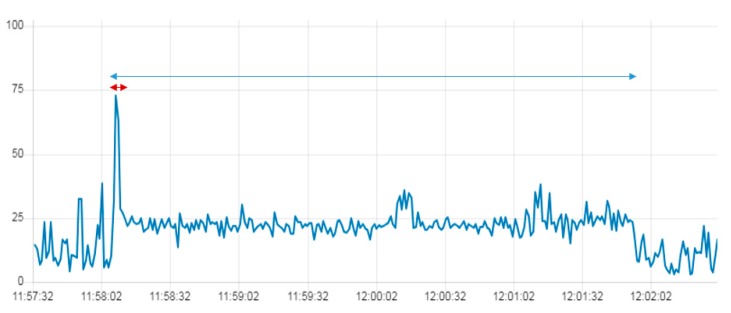
CPU Load while taking successive photos and writing them in a folder in the PC-G.

**Figure 24:**
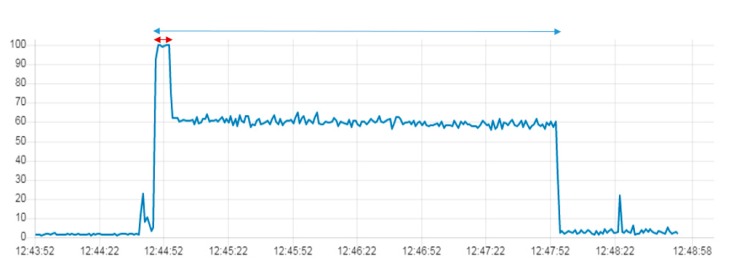
CPU Load while taking successive photos and writing them in a folder in the RPI-G.

**Figure 25:**
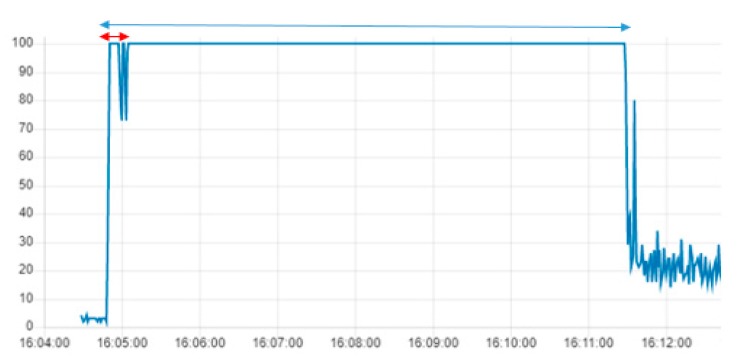
CPU Load while taking successive photos and writing them in a folder in the S-G.

Figure 23, Figure 24 and Figure 25 compare the CPU load of the same program implemented in the IoT gateways. The program continuously takes images from the drone and stores them in a file in the gateway. The first period (red interval) in all three graphs shows the connection between the drone and the gateways, while the rest of the period is the time of execution of the Node.js program in the gateways.

The three IoT gateways have different environmental specifications. Figure 23, Figure 24 and Figure 25 show these differences in terms of CPU usage in the three gateways while executing the mission. In RPI-G, the CPU load increases from 2% to 60%, while in S-G, it increases from 3 to 100%. In the PC-G gateway the average CPU load while executing the mission was around 20%. This difference is justified mainly by the numbers of cores implemented in each gateway processor. RPI-G used a 1.2 GHz Quad-Core ARMv7 processor with four cores, while S-G used a 400 MHz Intel Quark ×1020 processor with a single core. Furthermore, RPI-G and PC-G both support the Graphics Processing Unit (GPU), while S-G does not.

## 7. Conclusions

This paper introduces a model designed to monitor the smart industrial Internet of things based on an unmanned aerial vehicle, leveraging cloud computing services and using fog computing as a bridge between the different IIoT layers. The proposed model is able to monitor the condition of a concrete plant production line and the condition of the materials transported on conveyor belts to control the process. The results reveal the effectiveness of integrating drones with deep learning cloud services for processing and analyzing photos acquired in real-time. We demonstrate how to overcome the challenge of interoperability using fog and Node-RED computation on the IoT gateway. Node-RED interacts simultaneously with the different systems involved through different protocols. Drones now show great potential for many construction applications by reducing costs and improving production processes. Cloud services can handle many cases efficiently, although latency presents a major challenge due to the interaction between the different systems. The period of time available to the control system to decide and adjust the formula is estimated at between 38 and 60 s, depending on the quantity ordered by the customer and the composition of the formula. Given these points, the 3 s latency of the proposed solution is acceptable for plant control decisions.

The Siemens IoT gateway S-G is expected to provide better performance in an industrial setting, although it has less capacity than RPI-G. The RTD differences between S-G and RPI-G were caused by the CPU load in each machine, which reached 100% of S-G by connecting to the UAV. The IoT gateway provides an efficient solution for data communication, although the environmental specification of each IoT gateway is crucial when it comes to applications that require good computing performance.

## Figures and Tables

**Table 1:** Confusion matrix.

	Predictive Positive	Predictive Negative
**True Positive**	58 (*TP*)	6 (*FN*)
**True Negative**	9 (*FP*)	45 (*TN*)

**Table 2:** Specification of each machine environment.

	Siemens Gateway IOT2040	Raspberry Pi 3 Model B	Toshiba SATELLITE C870
**Ethernet**	2 × 10/100 Ethernet RJ45	10/100 BaseT Ethernet socket	10/100 BaseT Ethernet RJ-45
**Processor**	Intel Quark ×1020 400 MHz	1.2 GHz Quad-Core ARMv7	Intel Core i3 2348-M CPU 2.3 GHz
**Operation System**	Linux Kernel 4-4-18 Yocto Standard	Linux Raspbian 4.14.79-v7+	Windows 7 Professional
**RAM**	1 GB	1 GB	8 GB
**Disk Memory**	32 GB	16 GB	500 GB

**Table 3:** RTD test of 5200 samples from the OPC UA client to the OPC UA server (PLC) over different clients through different machines.

Client Test Environment	Data Type	Average	Standard Deviation	Minimum Latency	Maximum Latency
S-G	BOOL (1 bit)	23.160 ms	23.56 ms	19 ms	878 ms
RPI-G	BOOL (1 bit)	8.22 ms	3.48 ms	5 ms	76 ms
PC-G	BOOL (1 bit)	3.288 ms	2.65 ms	0 ms	32 ms

**Table 4:** RTD test of 100 samples from the IoT gateway to IBM Watson over different machines.

Client Test Environment	Average	Standard Deviation	Minimum Latency	Maximum Latency
S-G	1913.18 ms	522.17 ms	1454 ms	5594 ms
RPI-G	1373.09 ms	453.64 ms	1080 ms	5151 ms
PC-G	1129.29 ms	181.97 ms	980 ms	2491 ms

**Table 5:** SpeedTest over the 3 gateways.

Machine	Ping	Download	Upload
S-G	169.4 ms	16.3 Mbps	9.5 Mbps
RPI-G	96.4 ms	17.6 Mbps	13.8 Mbps
PC-G	55.7 ms	17.5 Mbps	12.3 Mbps

**Table 6:** RTD Test of 200 photos sent from the IoT gateway to the AR.drone 2.0.

Client Test Environment	Connection Established	Average	Standard Deviation	Minimum Latency	Maximum Latency
S-G	11429 ms	1229.92 ms	365.71 ms	160 ms	2906 ms
RPI-G	5348 ms	317.76 ms	411.18 ms	12 ms	1706 ms
PC-G	4562 ms	132.72 ms	35.90 ms	4 ms	230 ms
